# Comparative analysis of lysyl oxidase (like) family members in pulmonary fibrosis

**DOI:** 10.1038/s41598-017-00270-0

**Published:** 2017-03-10

**Authors:** Verena Aumiller, Benjamin Strobel, Merrit Romeike, Michael Schuler, Birgit E. Stierstorfer, Sebastian Kreuz

**Affiliations:** 1 Immunology & Respiratory Diseases Research, Boehringer Ingelheim Pharma GmbH & Co. KG, Biberach an der Riss, Germany; 2 Target Discovery Research, Boehringer Ingelheim Pharma GmbH & Co. KG, Biberach an der Riss, Germany

## Abstract

Extracellular matrix (ECM) composition and stiffness are major driving forces for the development and persistence of fibrotic diseases. Lysyl oxidase (LOX) and LOX-like (LOXL) proteins play crucial roles in ECM remodeling due to their collagen crosslinking and intracellular functions. Here, we systematically investigated LOX/L expression in primary fibroblasts and epithelial cells under fibrotic conditions, Bleomycin (BLM) induced lung fibrosis and in human IPF tissue. Basal expression of all LOX/L family members was detected in epithelial cells and at higher levels in fibroblasts. Various pro-fibrotic stimuli broadly induced LOX/L expression in fibroblasts, whereas specific induction of LOXL2 and partially LOX was observed in epithelial cells. Immunohistochemical analysis of lung tissue from 14 IPF patients and healthy donors revealed strong induction of LOX and LOXL2 in bronchial and alveolar epithelium as well as fibroblastic foci. Using siRNA experiments we observed that LOXL2 and LOXL3 were crucial for fibroblast-to-myofibroblast transition (FMT). As FMT could only be reconstituted with an enzymatically active LOXL2 variant, we conclude that LOXL2 enzymatic function is crucial for fibroblast transdifferentiation. In summary, our study provides a comprehensive analysis of the LOX/L family in fibrotic lung disease and indicates prominent roles for LOXL2/3 in fibroblast activation and LOX/LOXL2 in IPF.

## Introduction

Idiopathic pulmonary fibrosis (IPF) is a lethal chronic lung disease characterized by excessive accumulation of extracellular matrix (ECM), finally leading to disrupted lung architecture and severely impaired lung function. Although the origin of IPF is still unclear, uncontrolled wound repair caused by repetitive micro-injuries of the lung epithelium and resulting aberrant epithelial cell - fibroblast crosstalk is thought to be a main cause of IPF^1, 2^. Thus far, treatment options for IPF are limited as therapies which restore tissue homeostasis do not yet exist. In recent years, the post-transcriptional modification of ECM components has become a major focus of fibrosis research. Mechanical stress caused by increased crosslinking and stiffening of ECM is now regarded as a crucial factor for the self-perpetuating nature of IPF and other fibrotic disorders^3^. In the lung, ECM components constitute the interstitial connective tissue matrix, which has its major function in scaffolding tissue but also serves as a local reservoir for growth factors like FGF, WNT ligands and most importantly for tissue fibrosis, TGF-β^4^. By exceeding storage and release of these pro-fibrotic growth factors, the ECM plays a pivotal role in the regulation of fibrosis-relevant signaling pathways. In addition, the fibrotic ECM itself can stimulate fibroblasts to increase ECM production by a mechanism often referred to as mechanotransduction, thus representing a pro-fibrotic positive feedback loop^5, 6^.

Fibroblasts play a central role in building, maintaining and remodeling of ECM. Under physiological conditions, fibroblasts produce collagens, elastins and glucosaminoglycans, but also ECM-modulating proteases like MMPs. Under fibrotic conditions, fibroblasts trans-differentiate into myofibroblasts mainly induced by TGF-β. Activated myofibroblasts contribute to disease progression by excessive production of ECM^7^ with collagen I being the major component^8^. Moreover, myofibroblasts are characterized by elevated expression of α-smooth muscle actin resulting in stress fiber formation and increased contractibility.

The biosynthesis of collagen is tightly controlled on the transcriptional level and by post-transcriptional modifications including glycosylation, covalent crosslinking and proteolytic cleavage. Crosslinking of collagen and elastin via oxidation of lysyl amine residues is the final step in ECM maturation and is mediated by members of the lysyl oxidase family^9^. Lysyl oxidases (LOX) belong to the family of lysine-tyrosylquinone (LTQ)-dependent copper amine oxidases. The LOX/L protein family consists of five members including the prototypic LOX, and four subsequently discovered LOX-like paralogs LOXL1, LOXL2, LOXL3 and LOXL4. Structurally, all proteins share a conserved C-terminal catalytic domain including a His-X-His-X-His copper binding motif and a LTQ cofactor. At the N-terminus, LOX and LOXL1 share a basic secretion signal and a pro-form cleavage site for BMP1. In contrast to LOX and LOXL1, the remaining family members contain four scavenger receptor cysteine rich (SRCR) domains in their N-terminal domains^10^ of yet unknown function. LOX/L proteins are expressed in various tissues and their dysregulation is associated with the pathology of several diseases including cancer and fibrotic diseases^11–13^.

Of all LOX/L family members, so far LOX and LOXL2 have been studied most extensively. In addition to the well-known extracellular function as ECM-modifying enzymes, studies also suggest intracellular functions for both enzymes^14^. Particularly, it was demonstrated that LOXL2 is associated with epithelial to mesenchymal transition (EMT) by mediating Snail-dependent down-regulation of e-cadherin in human breast cancer cells^15, 16^. Furthermore, it has been observed that intracellular LOXL2 can modulate heterochromatin formation by deamination of H3K4, thereby influencing Snail transcription and inducing EMT^17^. Additionally, one study showed that LOXL2 is a negative regulator of Notch1 transcription, thereby attenuating epidermal differentiation^18^.

Studies demonstrating therapeutic efficacy of a LOXL2-specific antibody in experimental lung fibrosis models point towards a prominent role of LOXL2 in fibrotic remodeling^19^. The role of LOXL2 in fibrotic diseases is further supported by a study of Chien and co-workers, in which a positive correlation of LOXL2 serum levels with disease severity and progression was described in IPF patients^20^. Also LOX serum levels are associated with fibrotic diseases and are suggested as biomarker for systemic sclerosis^21^. However, a recent phase II clinical study in IPF using the LOXL2 specific antibody Simtuzumab was not able to show significant improvement of the respective endpoints^22^, raising the possibility that sole targeting of LOXL2 might not be sufficient. Likewise, even though there is evidence that lysyl oxidases play important roles in pathological processes associated with tissue fibrosis, the contribution of the individual LOX/LOXL family members to ECM remodeling, fibroblast activation and EMT is not fully understood. Therefore, to deepen our understanding of the function of LOX/LOXL in pulmonary fibrosis, we performed a systematic comparison of the LOX/LOXL family members by combining expression analyses in lung tissue samples and primary human lung cells with functional siRNA studies in human lung fibroblasts.

## Results

### Pro-fibrotic stimuli induce alterations of LOX/L gene expression in primary human lung fibroblasts and airway epithelial cells

**Figure 1:**
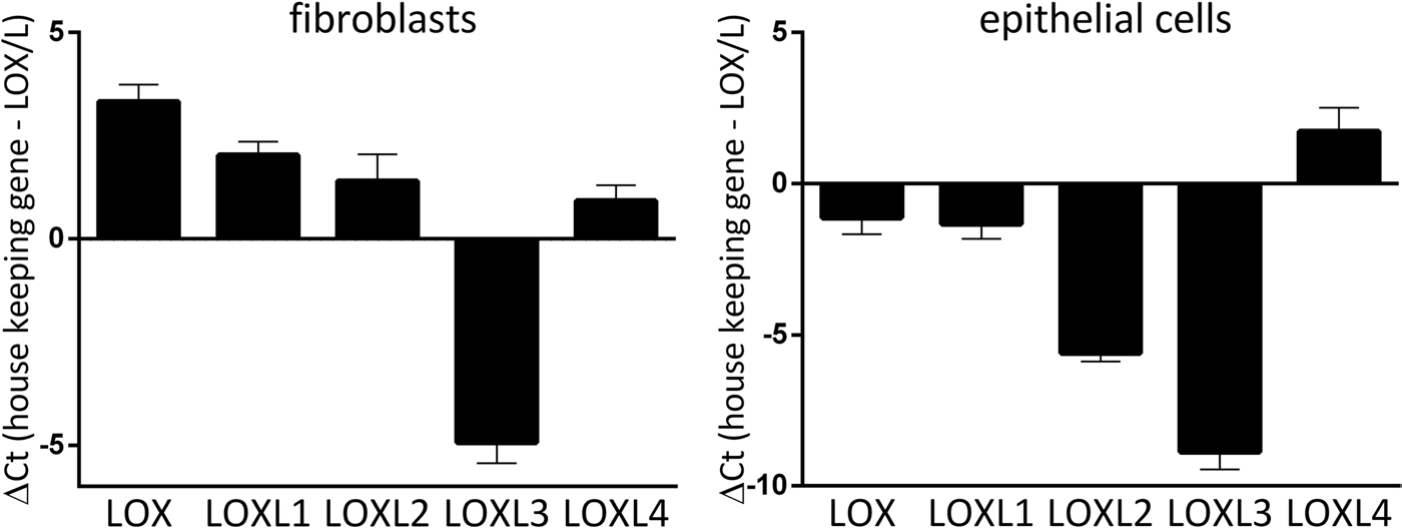
Basal expression levels of lysyl oxidases in primary human lung cells. LOX/L gene expression in primary human lung fibroblasts (NHLF) (left) or in air-liquid-differentiated primary bronchial epithelial cells (HBEC) (right) was determined using Taqman gene expression assays. Data is normalized to *RNA-polymerase 2* expression (Ct value of 25) and presented as mean of ΔCt ± SD of five independent experiments.

In order to assess LOX/L function in fibrosis-relevant cellular systems, we first analyzed the basal gene expression pattern of the individual family members in primary human lung cells. Figure 1 indicates LOX/L levels in normal human lung fibroblasts (NHLFs) and differentiated primary human bronchial epithelial cells (HBECs) relative to RNA-polymerase-II expression. We detected basal expression of all members in both cell types. In NHLFs, LOX was the highest expressed (ΔCt value of 3.2) followed by LOXL1 (ΔCt 2.0), LOXL2 (ΔCt 1.4) and LOXL4 (ΔCt 0.9). In HBECs, LOXL4 was the highest expressed gene (ΔCt 1.7), followed by LOX (ΔCt −1.1), LOXL1 (ΔCt −1.3) and LOXL2 (ΔCt −5.6). For LOXL3 we observed the lowest basal expression in NHLFs (ΔCt −4.9) and HBECs (ΔCt −8.9).

**Figure 2:**
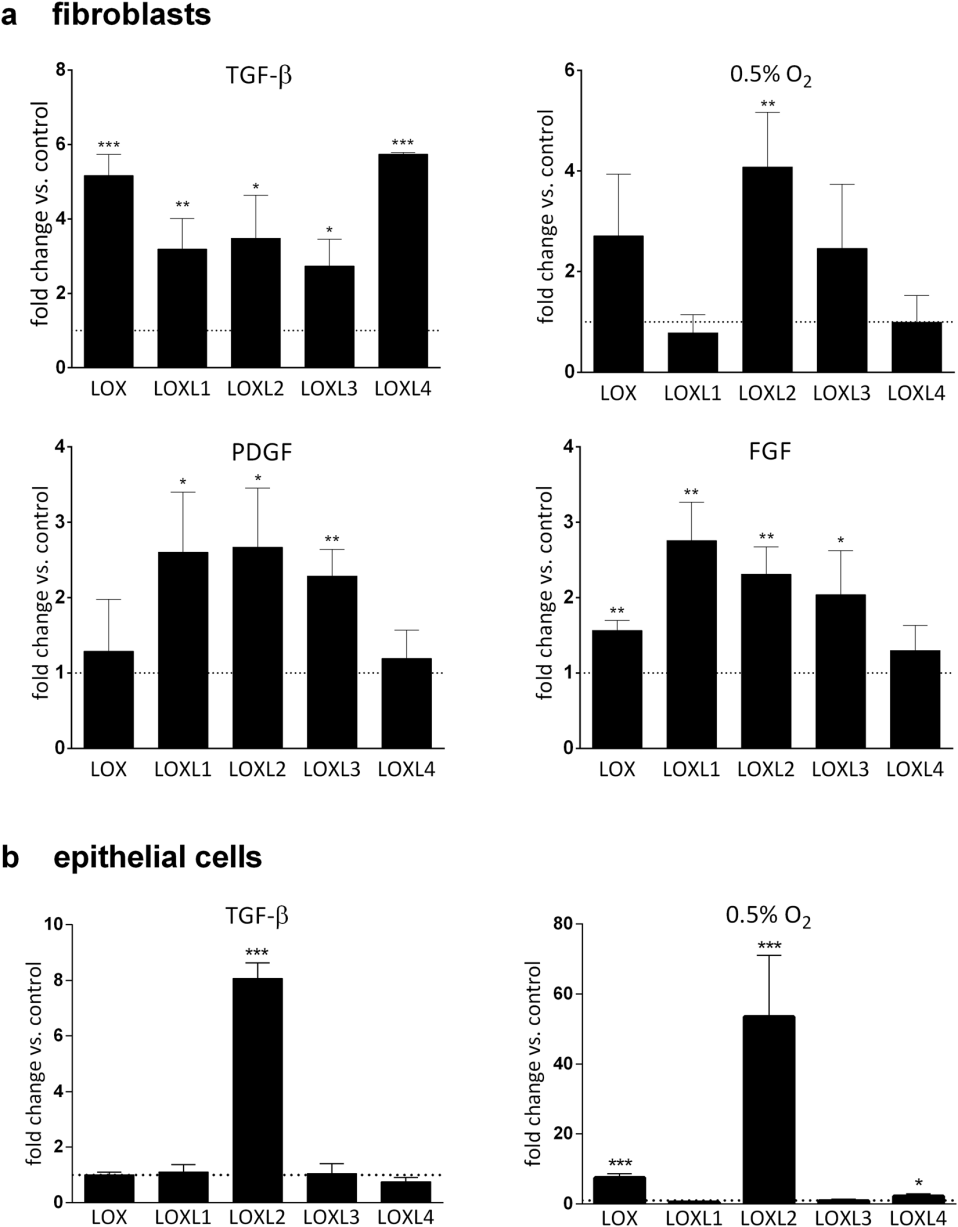
LOX/L gene expression level changes induced by various pro-fibrotic stimuli in primary human lung cells. (**a**) Primary human lung fibroblasts (NHLF) were treated with TGF-β1 (5 ng/ml), FGF (20 ng/ml) or PDGF (50 ng/ml) for 24 hours or kept under hypoxic conditions (0.5% O_2_) for 24 h followed by RNA isolation and quantitative PCR for the members of the LOX/L family. Data was normalized to RNA polymerase 2 expression and depicted as fold change relative to respective control treatment. Data is shown as mean ± SD of 3–4 independent experiments. (**b**) Primary human bronchial epithelial cells (HBECs) were differentiated at the air liquid interface for 21 days followed by basal treatment with TGF-β1 (5 ng/ml) for 48 h or kept under hypoxic conditions for 24 hours. RNA was isolated and quantitative PCR for the members of the LOX/L family was performed. Expression data was normalized to RNA polymerase 2 expression and control treatment and is depicted as mean ± SD of three experiments. *p < 0.05; **p < 0.01; ***p < 0.001, relative to control treatment.

To analyze LOX/L expression in a fibrotic context, we next stimulated NHLFs and HBECs with TGF-β, FGF and PDGF or kept them under hypoxic conditions (0.5% O_2_) (Fig. 2). In NHLFs, TGF-β significantly induced expression of all lysyl oxidase genes with fold changes ranging from 2.7 to 5.7 (stimulated vs. control). We observed a similar induction pattern for FGF and PDGF. LOXL1, LOXL2 and LOXL3 expression was increased with fold changes ranging from 2.0 to 2.7 (stimulated vs. control), while LOX and LOXL4 seemed not to be inducible by FGF and PDGF. Under hypoxic conditions, LOX, LOXL2 and LOXL3 levels were significantly elevated in NHLFs (2.5–4.1 fold under hypoxic vs. normoxic conditions). Interestingly, while gene expression of LOXL1-3 in NHLFs was induced by several stimuli, LOXL4 was only inducible by TGF-β (5.7-fold stimulated vs. control). In contrast to the rather broad induction pattern of LOX/L genes observed in NHLF cells, we found more distinct gene expression changes in HBECs. Remarkably, LOXL2 was the only family member showing pronounced upregulation after TGF-β stimulation (8.1-fold compared to unstimulated control). Under hypoxic conditions (0.5% O_2_) we observed a strong induction of LOXL2 expression (53.4-fold vs. normoxia) and a significant but less pronounced induction of LOX expression (7.5-fold vs. normoxia).

### LOX/L family members are differentially expressed in experimental lung fibrosis and human IPF lungs

**Figure 3:**
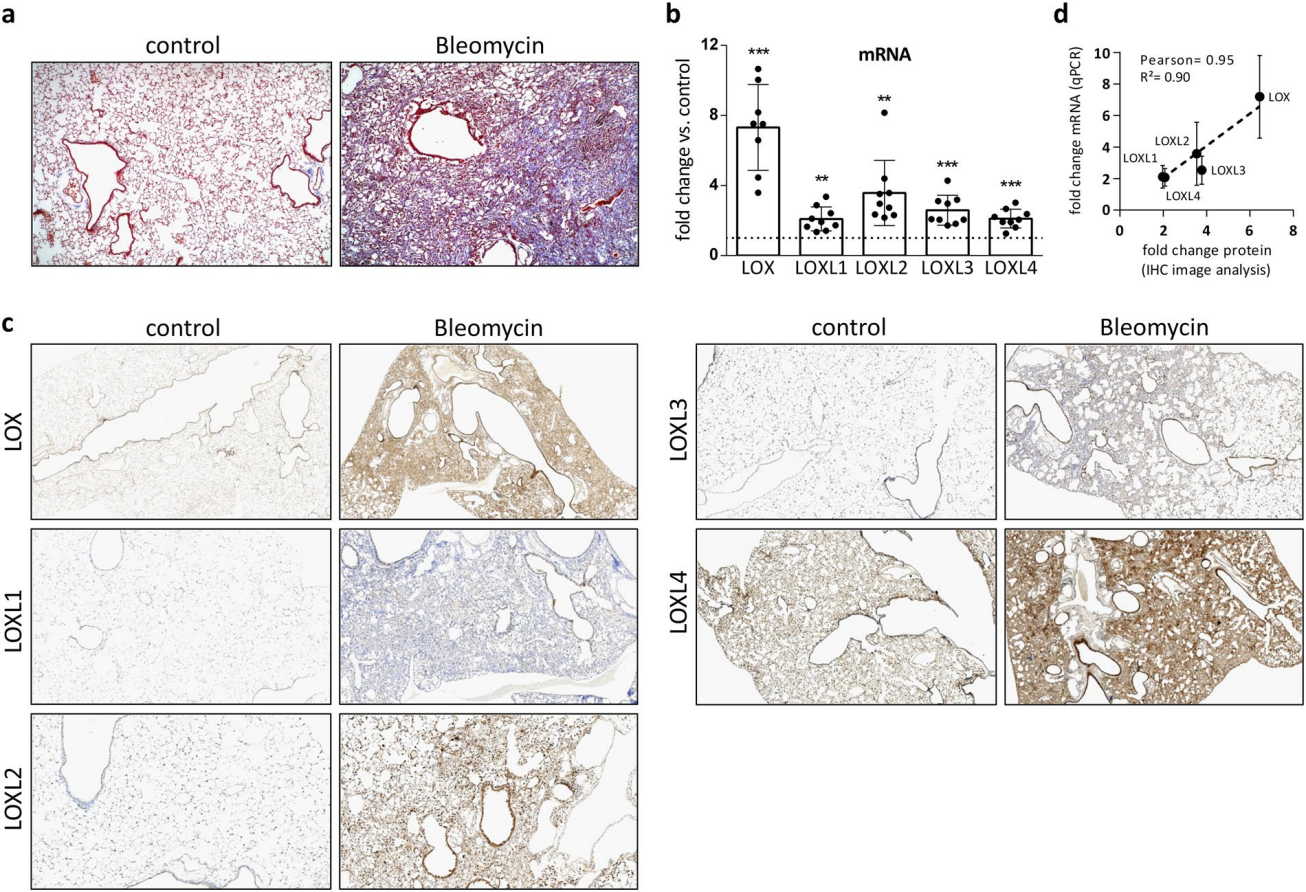
LOX/L expression in experimental lung fibrosis. (**a**) Masson trichrome stained histological sections of control (NaCl) or Bleomycin treated animals, 14 days after Bleomycin instillation. Nuclei appear in dark red, cytoplasm in light red and collagen in blue. (**b**) LOX/L gene expression was determined in whole lung homogenates from Bleomycin treated mice at day 14 after treatment using Taqman gene expression assays. Data is normalized to RNA-polymerase 2 expression (Ct value of 25) and presented as mean fold change ± SD of 8–9 animals per group. *p < 0.05; **p < 0.01; ***p < 0.001, relative to control treatment. (**c**) Representative images of immunohistochemical analyses of LOX, LOXL1, LOXL2, LOXL3 and LOXL4 at day 14 after intratracheal Bleomycin instillation. Positive cells are depicted in brown. (**d**) Correlation of the mRNA results shown in (**b**) with computational IHC image analysis-derived fold changes in protein expression (see Supplemental Fig. S2 for details).

In order to further validate our findings from the *in vitro* expression experiments, we next analyzed LOX/L RNA and protein expression in the Bleomycin (BLM) induced lung fibrosis model. Figure 3a shows Masson trichrome stained lung slices of mice 14 days after intratracheal (i.t.) Bleomycin instillation, illustrating fibrosis development and strongly increased collagen deposition (blue staining). In Fig. 3b, gene expression changes of the respective *LOX/L* mRNAs were determined by qPCR. *LOX* and *LOXL2* mRNA showed the highest induction (7.3 fold and 3.6 fold vs. PBS treated control mice, respectively), whereas upregulation of LOXL1, 3 and 4 was less pronounced (2.1, 2.6 and 2.1 fold vs. PBS control, respectively). Figure 3c depicts immunohistochemistry of lung sections at day 14 after i.t. BLM instillation. We excluded antibody cross reactivity within the LOX/L family using NHLF siRNA knockdown-lysates and recombinant LOX, LOXL2 and LOXL3 protein in Western Blot analysis (see Supplementary Fig. S1).

In agreement with the mRNA data obtained, we found LOX and LOXL2 clearly induced after BLM treatment in immunohistochemistry analyses (Fig. 3c). Elevated expression for LOX and LOXL2 was present in epithelial and interstitial regions of the fibrotic lung. In contrast to LOX and LOXL2, LOXL1 and LOXL3 showed at best mildly elevated protein expression in BLM-treated mice compared to control animals. LOXL4 showed high basal expression in the bronchial and alveolar epithelium, macrophages and fibroblasts, with a significant further increase upon BLM treatment. Notably, a very good correlation between the expression changes observed on the mRNA level (Fig. 3b) and protein level (Fig. 3c) was observed, when we compared qPCR data with data derived from computational IHC image analysis (Fig. 3d and Supplementary Fig. S2, for processed images and a detailed description of methodology).

**Figure 4:**
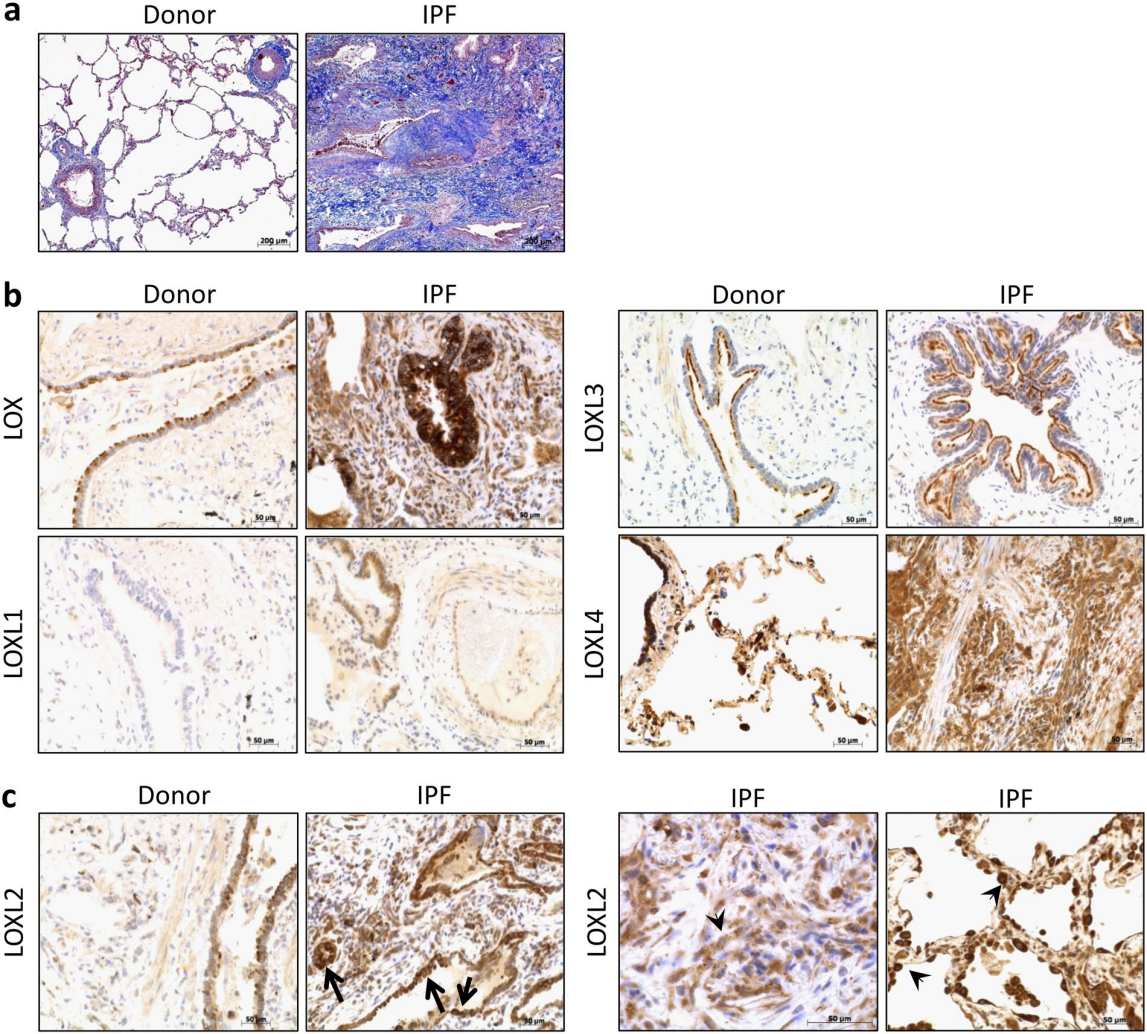
LOX/L expression in human IPF lungs. (**a**) Masson trichrome stained histological sections of donor and IPF patient lung tissue. Nuclei appear in dark red, cytoplasm in light red and collagen in blue (scale bar = 200 ${\mu }{\rm{m}}$). (**b**) Expression of LOX, LOXL1, LOXL3 and LOXL4 in IPF and donor lungs. Representative pictures of immunohistochemistry in tissue specimen of patients with IPF and transplant donors are shown. Positive cells are depicted in brown (scale bar = 50 ${\mu }{\rm{m}}$). (**c**) Immunohistochemistry of LOXL2 in tissue specimen of patients with IPF and transplant donors. LOXL2 in bronchial epithelium is marked with black arrows. LOXL2 expression in hyplerplastic alveolar epithelial cells in the interstitial region and fibroblast foci are indicated by arrow heads (scale bar = 50 ${\mu }{\rm{m}}$).

To link our findings to the human disease, we next analyzed human lung sections of five healthy donors and 14 IPF patients with regard to LOX/L expression. Figure 4a shows Masson trichrome stained tissue sections, clearly demonstrating IPF pathology and massive deposition of collagen in affected areas (blue staining). As shown in Fig. 4b and c, we observed basal expression of LOX, LOXL2 and LOXL4 in lung tissue samples of healthy patients with bronchial epithelial cells being the main sites of expression. In IPF, similar to our findings in the BLM model, LOX and LOXL2 protein expression was clearly elevated in epithelial cells compared to healthy controls; moreover, increased expression levels were detected in the lung interstitium. Notably, LOXL2 was the only LOX/L family member showing distinct staining in fibroblastic foci and increased protein expression in alveolar epithelial cells (Fig. 4c). In contrast to LOX and LOXL2, only moderately elevated expression compared to the basal levels was observed for LOXL1 and LOXL4 in IPF lungs with bronchial epithelium, fibrotic foci and to a lesser extent large vessel walls being the main sites of expression (Fig. 4b). Interestingly, we also observed a high variability of LOXL1 expression levels between different IPF donors (data not shown). Immunohistochemistry analysis of LOXL3 revealed a specific expression pattern restricted to the cilia of bronchial epithelial cells in normal as well as IPF lungs. To exclude unspecific binding of the LOXL3 antibody, experiments using a blocking peptide for the LOXL3 epitope were performed and specificity of the LOXL3 antibody was confirmed (see Supplementary Fig. S1). In summary, our findings point towards a differential expression pattern of LOX/L family members in fibrotic tissue remodeling with LOX and LOXL2 showing the strongest up-regulation under fibrotic conditions, whereas for the remaining LOX/L family members only mild (LOXL1 and LOXL4) or unchanged (LOXL3) expression levels were observed. Our results are summarized in Table 1.

**Table 1:** LOX/L family protein expression in human lung tissue.

	Healthy lung		IPF lung	
LOX	++++	Cilia of bronchial epithelium	++++	Bronchial epithelium
	+	Connective tissue	+++	Fibrotic foci
			+++	Hyperplastic alveolar epithelial cells
LOXL1	−		+ to ++	Bronchial epithelium
			+ to ++	Fibrotic foci
			+	Hyperplastic alveolar epithelial cells
			+	Large vessel walls
LOXL2	+++	Bronchial epithelium	++++	Bronchial epithelium
	++	Alveolar epithelium	+++	Fibrotic foci
	++	Pneumocytes/Macrophages	++++	Hyperplastic alveolar epithelial cells
LOXL3	++++	Cilia of bronchial epithelium	++++	Cilia of bronchial epithelium
LOXL4	+++	Bronchial epithelium	+++	Bronchial epithelium
	+++	Alveolar epithelium	+++	Alveolar epithelium
	+++	Pneumocytes/Macrophages	+++	Pneumocytes/Macrophages
	+++	Vascular endothelium	+++	Vascular endothelium
			+ to ++	Fibrotic foci

### LOX/L family members are required for fibroblast to myofibroblast transition (FMT) in primary human lung fibroblasts

**Figure 5:**
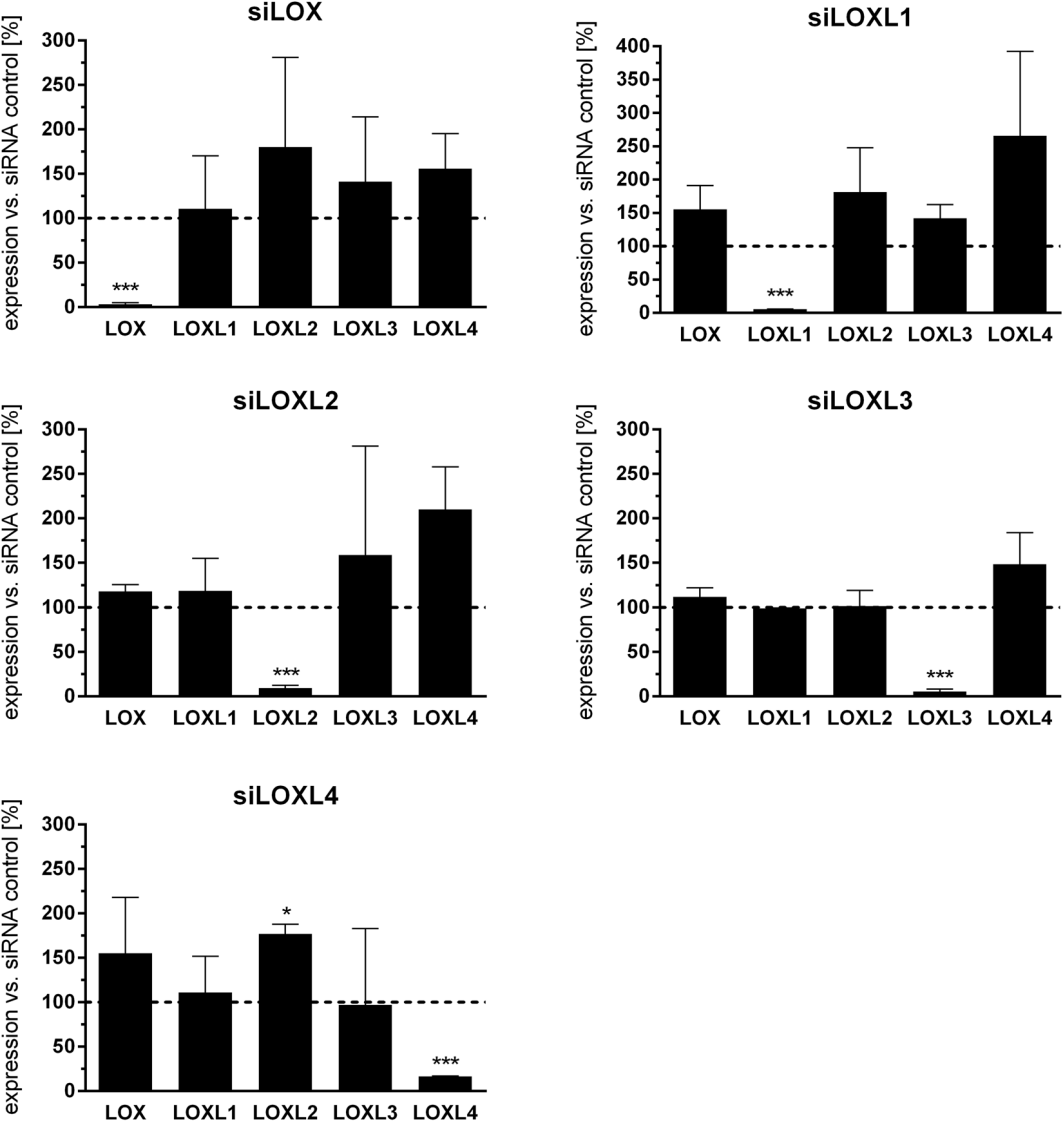
Knockdown efficacy and specificity of siRNA pools. Individual LOX/L siRNA pools were analyzed for their knockdown specificity following transfection of primary human lung fibroblasts (NHLFs) with 16.6 nM of control, LOX, LOXL1, LOXL2, LOXL3 or LOXL4 siRNA. 24 hours after transfection, mRNA levels of the individual LOX/L family members were determined using Taqman gene expression assays. The data is depicted as % expression compared to control siRNA treatment (which was set 100%) and presented as mean ± SD of two to four independent experiments. ***p < 0.001, relative to control.

After having identified differential expression patterns of LOX/L family members in experimental and human IPF-derived tissue samples, we next explored the individual contribution of LOX/L family members to fibroblast to myofibroblast transition (FMT), a key process during fibrotic remodeling. To this end, NHLF cells were transfected with LOX/LOXL-specific siRNAs 24 h prior to TGF-β stimulation. As a marker for FMT induction, *acta2* (i.e. α-smooth muscle actin) expression was measured 48 hours post TGF-β stimulation by qPCR. Analysis of knockdown (KD) efficiency and selectivity within the LOX/LOXL family revealed selective and efficacious KD for all siRNA pools used. Importantly, the KD of individual LOX/L variants did not induce significant alterations in the expression of other LOX/L variants, arguing against potential compensatory mechanisms (Fig. 5).

**Figure 6:**
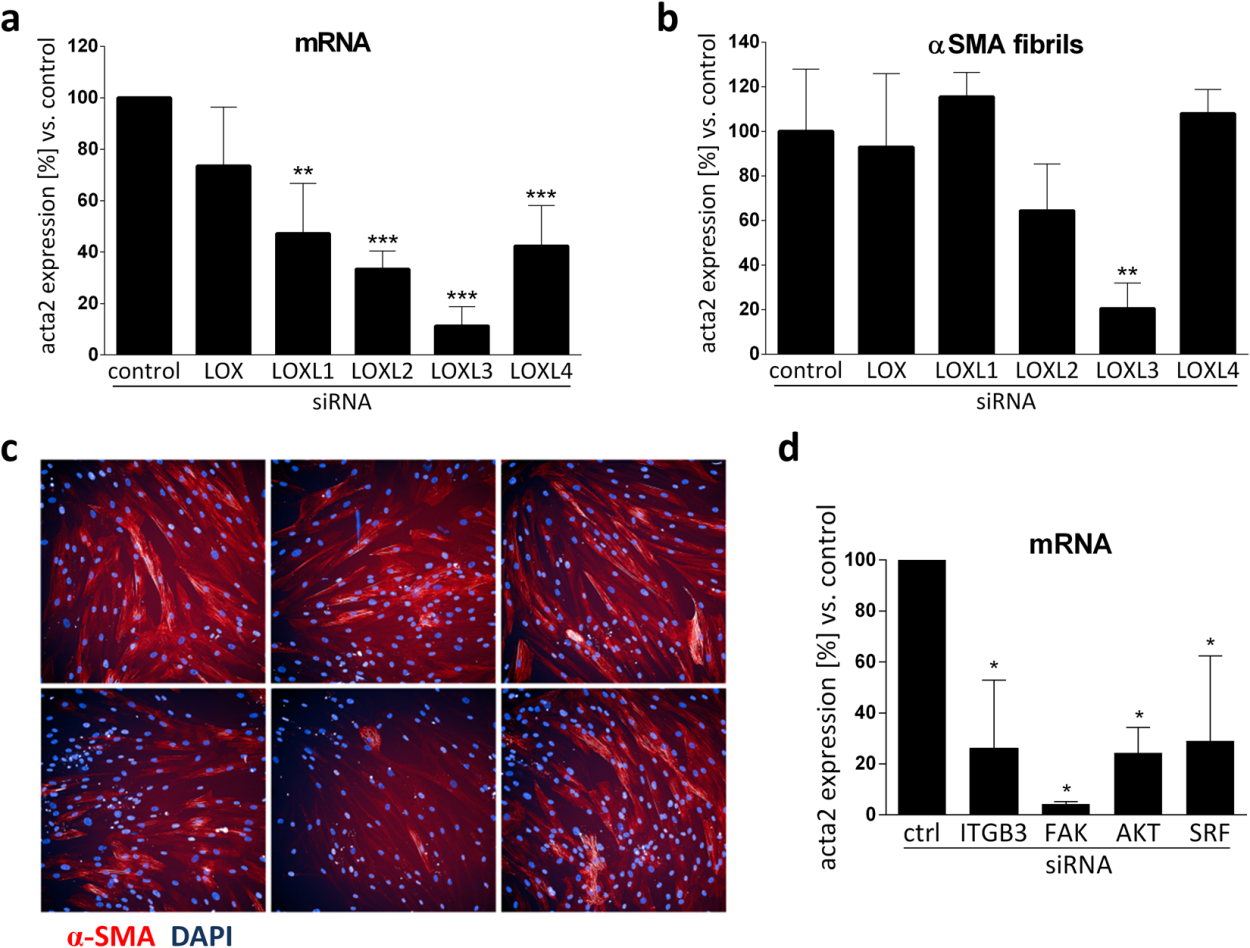
LOX/L knockdown leads to decreased smooth muscle actin production and stress fibre formation in NHLFs. (**a**) Primary human lung fibroblasts (NHLFs) were transfected with 16.6 nM of control, LOX, LOXL1, LOXL2, LOXL3 or LOXL4 siRNA. After 24 h, cells were stimulated with TGF-β (5 ng/ml) for 48 hours. Gene expression changes of *acta2* were determined using Taqman gene expression assays and normalized to RNA-polymerase 2 expression and control treatment. The data represent the mean ± SD of three independent experiments (**p < 0.01; ***p < 0.001). (**b**) NHLFs were transfected as described in (**a**) and stimulated with TGF-β1 for 72 hours. αSMA fibrils per cell were quantified after immunocytochemical staining using high-content cellular imaging of 16 wells of a 384-well plate per condition (see “Image analysis” in the methods section for details). The data represent the mean ± SD of three independent experiments (**p < 0.01). Representative pictures of the cells stained for αSMA (red) and nuclei (DAPI, blue) are shown in (**c**). (**d**) *acta2* induction by TGFβ1 on RNA level is decreased after FAK signaling pathway depletion. NHLF were transfected with 16.6 nM of control, ITGB3, FAK, AKT or SRF siRNA. After 24 h cells were stimulated with TGF-β1 (5 ng/ml) for 48 hours. Gene expression changes of acta2 were determined using Taqman gene expression assays. Data was normalized to RNA-polymerase 2 expression and control treatment and is presented as mean ± SD of three independent experiments (*p < 0.05).

KD of LOXL1, 2, 3 and 4 significantly decreased *acta2* mRNA levels (Fig. 6a). The strongest effects were observed for LOXL2 and LOXL3 with 70% and 86% decreased *acta2* gene expression, respectively, as compared to an unspecific siRNA control. Less prominent but still significant effects were observed for LOXL1 and 4 with 46% decreased *acta2* levels compared to control siRNA. Surprisingly, in contrast to the LOXL family members, KD of LOX had no effect on TGF-β induced *acta2* expression. We further confirmed our findings on protein level by quantifying the number of α-smooth muscle actin (α-SMA) fibrils per fibroblast following immuno-fluorescent α-SMA staining, by using a semi-automated high-content analysis script (Fig. 6b and c; see method section for details). Figure 6b shows the residual percentage of α-SMA fibril formation after LOX/L depletion and TGF-β stimulation compared to control siRNA. KD of LOXL2 and LOXL3 led to a 36% and 80% decrease in α-smooth muscle actin fiber formation, respectively. Representative fluorescent images of one out of 16 analyzed wells per condition, showing α-smooth muscle actin fibers in red and nuclei in blue are shown in Fig. 6c (additional images are available in Supplementary Fig. S3). Moreover, we exemplarily assessed an additional marker for activated fibroblasts by measuring collagen 1α expression in siLOXL2 transfected, TGFβ1 stimulated NHLFs. The results demonstrate that following LOXL2 KD, *col1a1* expression was reduced in a similar manner as *acta2* (see Supplementary Fig. S4). In summary, our data point towards a role of LOXL2-4 during TGF-β–mediated FMT in NHLFs, whereas LOX seems to be dispensable for fibroblast activation.

In cancer-associated fibroblasts (CAF), LOXL2-mediated crosslinking of ECM triggers transcriptional changes via focal adhesion kinase (FAK)-dependent mechano-transduction^23^, resulting in fibroblast activation and αSMA production. In order to analyze whether FMT induction in lung fibroblasts is also dependent on a similar mechanism, we used siRNAs to knock down genes within the FAK pathway, including integrin-β3 (ITGB3), focal adhesion kinase (FAK), protein kinase B (AKT) and serum response factor (SRF) and analyzed *acta2* mRNA expression to assess FMT induction. After having confirmed the KD efficiency and specificity of the siRNA pools used (see Supplementary Fig. S5), NHLFs were transfected with the respective siRNAs followed by TGF-β stimulation. KD of ITGB3, FAK, AKT and SRF led to significantly decreased *acta2* induction ranging from 71% to 96% inhibition (Fig. 6d), thereby clearly indicating a role of FAK-dependent mechano-transduction during FMT in human lung fibroblasts, in analogy to earlier findings in cancer. Interestingly, similar to TGFβ1, stimulation of naïve NHLF cells with recombinant LOXL2 led to an increase in total and phospho-AKT (see Supplementary Fig. S6), thereby supporting the hypothesis that LOXL2 might drive fibroblast activation through this pathway.

### LOXL2-mediated effects on FMT are dependent on its enzymatic activity

**Figure 7:**
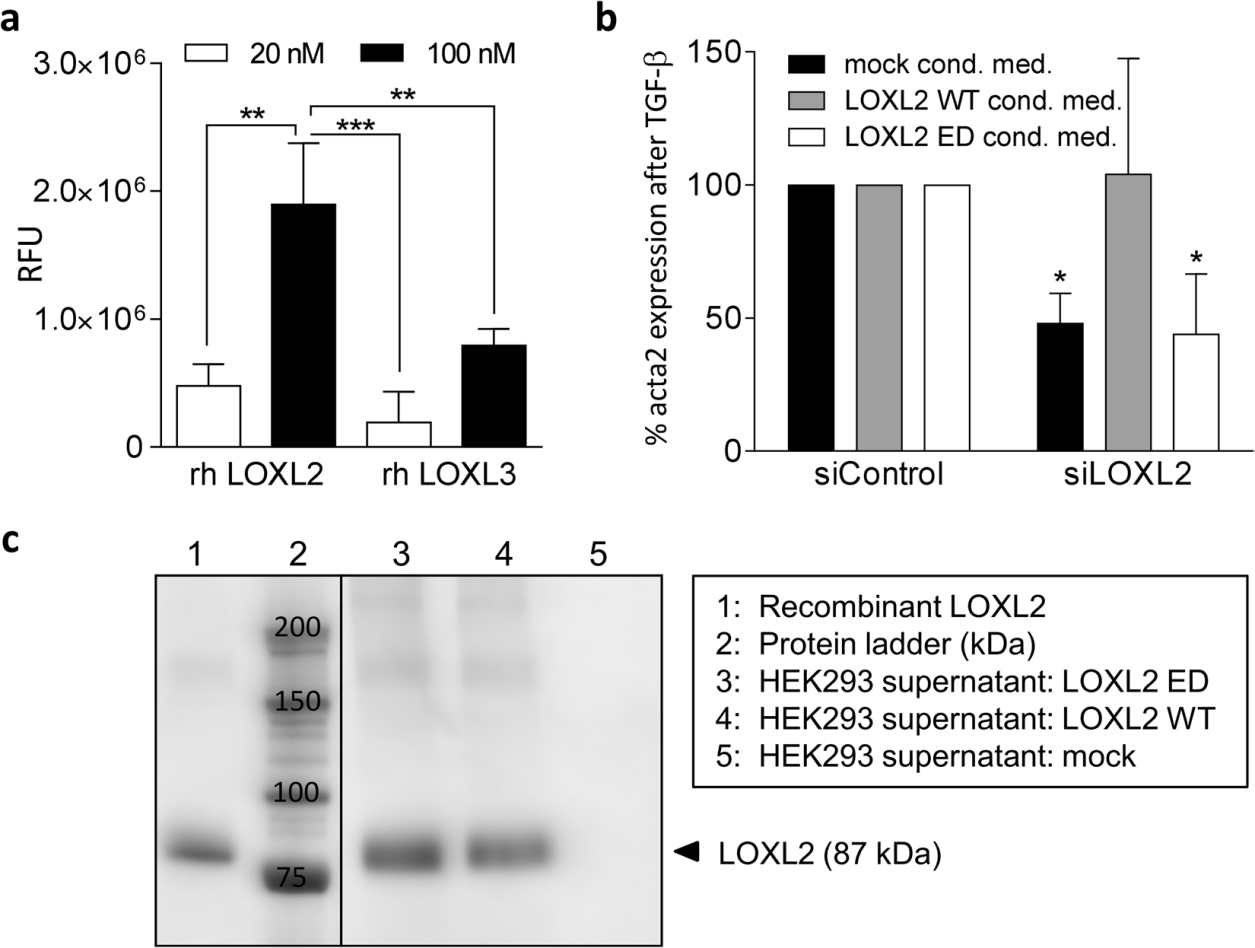
FMT is dependent on the enzymatic activity of LOXL2. (**a**) Comparison of collagen I crosslinking capacity of recombinant LOXL2 and LOXL3. Human lung collagen I coated microplates were incubated with LOXL2 or LOXL3, respectively, for 2 hours followed by aldehyde detection using O-(biotinylcarbazoylmethyl) hydroxylamine as a probe. Relative fluorescence units (RFU) indicate the amount of aldehyde intermediate in collagen I produced by LOXL2 and LOXL3, respectively. White bars indicate resulting fluorescence after incubation with 20 nM LOX/L, whereas black bars represent 100 nM of the respective enzyme. Data is shown as mean ± SD of three independent experiments. **p < 0.01; ***p < 0.001. (**b**) NHLF cells were transfected with 16.6 nM of control or LOXL2 siRNA. After 12 hours, cells were incubated in conditioned medium derived from HEK293 cells transfected with LOXL2 wild type, LOXL2 enzymatic dead or control plasmids (mock). 24 h after transfection, cells were stimulated with TGF-β1 (5 ng/ml) for 48 hours in presence of the respective conditioned medium. Gene expression changes of *acta2* mRNA were determined using Taqman gene expression assays. Data are normalized to RNA-polymerase 2 expression and control treatment and presented as mean ± SD of three independent experiments. *p < 0.05, relative to siControl treatment. (**c**) LOXL2 protein content detected by LOXL2-specific Western Blotting, 72 hours after HEK293 transfection with the respective plasmids (10 ${\mu }{\rm{l}}$ supernatant per lane).

As we observed the strongest inhibition of fibroblast activation after LOXL3 knockdown, we speculated that LOXL3 might have a higher ECM crosslinking activity as compared to LOXL2, and hence a more pronounced contribution to matrix stiffening and subsequent mechanical fibroblast activation. Therefore, to compare the collagen crosslinking activity of LOXL2 and LOXL3 we applied an aldehyde detection assay, which allows for the quantification of allysine-aldehydes, intermediate products during lysyl oxidase-mediated collagen crosslinking. The resulting RFUs resemble the amount of aldehyde intermediate in collagen I, which was converted by lysyl oxidase during the incubation period (Fig. 7a). Our results show that both human recombinant LOXL2 and LOXL3 were able to oxidize human lung collagen in a concentration-dependent way. Notably, we observed a 2-fold higher collagen oxidation capacity of LOXL2 compared to LOXL3, thereby disproving the hypothesis that LOXL3 shows particularly high crosslinking activity.

Based on the expression pattern of LOXL2 in IPF lungs, its high collagen crosslinking capacity, the *in vitro* expression data and its crucial role during fibroblast activation (also see the summary in Table 2), we finally investigated the functional role of LOXL2 in fibroblast activation in more detail. To analyze whether the enzymatic activity of LOXL2 is required for fibroblast activation, we generated two plasmids constitutively expressing either LOXL2 wild type (LOXL2-WT) or a point-mutated (Y689F) version of LOXL2 lacking enzymatic activity (LOXL2-ED)^24^. Next, we transfected HEK293 cells with the LOXL2 plasmids or a GFP plasmid control (mock) to obtain conditioned medium, which we used to reconstitute LOXL2 function in the previously described TGF-β fibroblast activation assay, analyzing *acta2* gene expression after siRNA-mediated LOXL2 knockdown. In line with our previous results, LOXL2 depletion again led to a 52% decrease of *acta2* expression (Fig. 7b). However, whereas the addition of LOXL2-ED conditioned medium had no effect (56% reduced *acta2* level), treatment of the cells prior and during TGF-β stimulation with the LOXL2-WT conditioned medium reconstituted *acta2* expression to 104%. Importantly, the amount of LOXL2 protein in the supernatants was determined by Western Blot analysis and was comparable in both conditioned media preparations used (Fig. 7c). We therefore conclude that LOXL2-mediated ECM modification is augmenting α-SMA production under pro-fibrotic conditions in lung fibroblasts, and that the enzymatic activity is required for this effect.

**Table 2:** Summary of LOX/L expression changes under fibrotic conditions and impact on FMT based on the results of this study.

Method/Assay/Sample		LOX	LOXL1	LOXL2	LOXL3	LOXL4
Gene expression	Fibroblasts (NHLF)	+++	−	+++	++	+
	Epithelial cells (HBECs)	−	−	+++	−	−
IHC	Mouse (Bleomycin)	+++	−	+++	+	+
	Human IPF	+++	+	+++	−	+
Impact on FMT (siRNA study)		−	+	++	+++	+

## Discussion

Excessive deposition and aberrant post-translational modification of ECM components are hallmarks of pulmonary fibrosis, resulting in mechanical stress and persistent activation of fibroblasts. ECM-modifying enzymes like lysyl oxidases are regarded as key drivers of fibrotic remodeling and are therefore considered attractive drug targets for various fibrotic indications, including IPF. To unravel the contribution of individual LOX/L family members to IPF pathology, we systematically analyzed lysyl oxidases by combining gene and protein expression analyses and functional siRNA studies.

As a first step we analyzed basal gene expression levels of LOX/L family members in primary lung fibroblasts (NHLFs) and differentiated bronchial epithelial cells (HBECs). Under physiological conditions we detected basal expression of all LOX/L family members at varying levels in both cell types analyzed, with LOXL3 being the lowest expressed gene. Overall, LOX/L basal expression levels were more pronounced in fibroblasts than in epithelial cells. Stimulation of NHLF cells with pro-fibrotic growth factors (FGF, PDGF and TGF-β) resulted in a broad response with elevated expression levels for all LOX/L family members, where LOXL4 showed the most pronounced upregulation by TGF-β among all LOX/L genes. These findings are in line with earlier studies, which showed that LOXL4 is a direct target of TGF-β signaling in aortic endothelial cells^25^. Interestingly, a more specific expression pattern was observed in differentiated HBECs, where LOXL2 was the only LOX/L member showing elevated expression levels upon TGF-β stimulation. In addition, hypoxic challenge of fibroblasts and epithelial cells resulted in specific gene expression changes of LOX/L family members. While LOX, LOXL2 and LOXL3 were moderately up-regulated in NHLFs, LOX and LOXL2 were strongly induced in epithelial cells, which is in line with earlier findings in hepatoma cells^26^, thereby demonstrating a link between lysyl oxidases and hypoxia in the lung. Notably, our observation of elevated LOXL2 expression levels in bronchial epithelial cells upon TGF-β stimulation and hypoxia points towards a potential contribution of lung epithelial cells to ECM remodeling.

In addition to primary lung cells, we further analyzed protein expression and cellular distribution of LOX/L family members by immunohistochemistry in lung tissue samples derived from both, BLM-induced lung fibrosis in mice and 14 human IPF patients. In contrast to lung tissue from control treated mice, LOX and LOXL2 were highly induced in the BLM-induced fibrosis model. These results were additionally supported by LOX/L gene expression data obtained from total lung RNA sequencing in the BLM model and a novel model of AAV-TGFβ1-induced fibrosis^27^ (data not shown). Notably, in both, the murine and human lung, LOX and LOXL2 were predominantly induced in bronchial and alveolar epithelial cells. The current understanding of the contribution of epithelial cells to IPF pathology is that they are initiating fibrosis due to continuously exaggerated repair processes^28, 29^. Our data now raise the possibility that the excessive production of ECM-remodeling enzymes by the epithelium might additionally drive the fibrotic processes beneath. Whether lysyl oxidases contribute to EMT in IPF, as it was already described for LOX and LOXL2 in cancer^15, 30^, needs to be investigated in future studies.

Besides its pronounced expression in lung epithelial cells, we also observed increased expression of LOXL2 in fibroblastic foci, along with increased levels of LOX and LOXL1. Fibroblastic foci are spots of activated, proliferating myofibroblasts, which are characterized by elevated expression of αSMA and the formation of αSMA-positive stress fibers. For LOXL2, a co-localization with αSMA, a marker for activated fibroblasts, was previously shown in colon adenocarcinoma^19^. Notably, high levels of αSMA are considered to be negatively associated with IPF patient survival^31^ and might serve as a biomarker for IPF. Having identified increased expression of LOX, LOXL1 and LOXL2 in fibroblast foci, we aimed to explore the function of the different LOX/L family members during fibroblast to myofibroblast transition. Therefore, we analyzed αSMA expression and αSMA stress fiber formation in TGF-β-stimulated primary fibroblasts on the mRNA and protein level following siRNA-mediated knockdown of LOX/L family members. In line with earlier studies performed in the context of cancer associated fibroblasts (CAFs)^23^, we were able to show significantly reduced αSMA and collagen 1α expression following knockdown of LOXL2. Interestingly, for the first time, we observed an even more pronounced effect for LOXL3, while the knockdown of LOXL1 and LOXL4 only had minor influence on αSMA expression.

The discrepancy between elevated LOX expression in IPF tissue and its minor role in our FMT experiments might suggest, that the performed *in vitro* fibroblast assays rather reflect an early remodeling state of fibrosis, whereas the samples from IPF patients represent the already established late stage disease. This hypothesis might also explain why we found a crucial role for LOXL3 in FMT in the *in vitro* experiments, whereas LOXL3 expression was limited to ciliated cells but absent in fibrotic foci, as demonstrated by our IHC analysis. Given that there is also evidence for LOXL3 in EMT during tumor progression^32^, further studies are required to decipher the exact role of LOXL3 in EMT and FMT and, whether there are temporal, disease stage-dependent differences in its function. Finally, to fully confirm LOXL2 and LOXL3’s proposed role in augmenting FMT by remodeling ECM, in-depth analyses of collagen structure and ECM rigidity following, for instance, RNAi-mediated LOX/L depletion should be carried out in future studies. Moreover, it has to be assessed in detail whether there is indeed a direct link between LOX/L expression, altered focal adhesion kinase signaling and FMT, as suggested by our and previous studies in cancer-associated fibroblasts^23^.

In our study, LOXL2 was identified as the most relevant lysyl oxidase over all assays and analyses performed. LOXL2-mediated effects can be dependent on either the actual lysyl oxidase activity or on other mechanisms, for example intracellular protein-protein interactions. In literature, the intracellular functions of LOXL2 are currently controversially discussed. Cuevas and colleagues showed that stable transfection of enzymatically inactive mutants of LOXL2 leads to induction of EMT in kidney epithelial (MDCK) cells, which is mediated by LOXL2 binding to and silencing of the E-cadherin promoter^33^. In contrast, using transiently transfected breast cancer cell lines (MCF7), Moon and colleagues showed that only intracellularly accumulated, enzymatically active LOXL2 induced EMT, but enzymatically inactive LOXL2 had no effect on the epithelial phenotype of the cells. They postulate that the amount and the intracellular localization of LOXL2 rather than its enzymatic activity are relevant for the induction of EMT^16^. Reconstitution experiments in our FMT assay using LOXL2 wild-type and enzyme dead variants showed that a rescue of LOXL2 function during fibroblast activation was only possible with conditioned medium containing catalytically active enzyme. Thus, we conclude that LOXL2-mediated mechanotransduction is driven by its enzymatic ECM-crosslinking activity.

While out of all lysyl oxidase-like isoforms LOXL2 stood out as the most significantly altered family member in our study, thereby supporting current efforts to target LOXL2 in fibrosis, an open question is whether additional targeting of LOX or other lysyl oxidase family members like LOXL3 in combination with LOXL2 would be a desirable strategy from a therapeutic point of view. Whereas we detected strongly elevated LOX expression in human IPF lungs, unlike LOXL2, no effects on FMT after LOX knockdown were observed. In contrast, inhibition of LOXL3 during fibroblast activation had beneficial effects, namely reducing stress fiber formation. A recent study in muscular fiber development identified LOXL3 as an enhancer and key regulator of integrin signaling, supporting the role of LOXL3 in pro-fibrotic signaling pathways^34^. Moreover, LOX inhibition was shown to be efficacious in early inflammatory phases of the Bleomycin-induced fibrosis model, suggesting that a LOX inhibitory component in an anti-fibrotic treatment approach could be beneficial^35^. Therefore, a detailed evaluation of the potential of dual LOX/LOXL2 or LOXL2/LOXL3 targeting should be conducted in future studies. However, in this regard, it should be taken into account that developmental defects (lung and cardiovascular system for LOX, cardiovascular defects for LOXL2 and palate and spine defects for LOXL3) have been described in respective knockout mice, finally leading to perinatal death of these animals^18, 36–38^. Therefore, for dual targeting approaches, a careful assessment of potentially improved efficacy and limited feasibility due to challenging safety profiles will be necessary.

In summary, we provide the first systematic comparison of lysyl oxidase expression in experimental and clinical pulmonary fibrosis and their functional involvement in fibroblast to myofibroblast transition *in vitro*. Our results point towards an outstanding role of LOXL2 among the other lysyl oxidase family members, which seem to have partial redundancy in fibrotic tissue remodeling. As summarized in Table 2, LOXL2 was the only family member showing significant effects in fibrotic tissue remodeling, fibroblast to myofibroblast transition and pronounced up-regulation of gene expression under fibrotic conditions *in vitro* and *in vivo*. Based on the comparative analysis of LOX/L family members, our data also provide a solid basis for future studies evaluating the potential of individual versus simultaneous targeting of LOX/L family members for the treatment of IPF.

## Methods

### Cells

NHLF cells from LONZA (Basel, Switzerland) (CC-2512) were cultured according to standard protocols in FBM Medium. HBECs from LONZA (CC-2540) were expanded in BEGM and differentiated at the air liquid interface according to the protocol from StemCell Technologies (Vancouver, Canada) in PneumaCult media for 21 days.

### siRNA experiments

Cells were transfected with 16.6 nM siRNA pool (GE Healthcare, Chalfont, UK, using RNAimax in OPTIMEM Medium (Life technologies, Carlsbad, CA). After 24 hours, knockdown efficiency was analyzed by qPCR. 12 hours after transfection cells were cultured in starvation medium for 12 hours followed by TGF-β1 (5 ng/ml, R&D Systems, Minneapolis, MN) or control stimulation for 48 hours. For gene expression analysis cells were lysed in RLT and lysates were stored at −80 °C until further analysis. For immune fluorescent protein analysis cells were transfected in 384 well format and fixed in 3.7% paraformaldehyde (PFA).

### Cell treatments

Cells were starved overnight in FBM + 0.1% FCS without supplements, then treated with either control, TGF-β1 (5 ng/ml), FGF (20 ng/ml), PDGF (50 ng/ml) or were kept under hypoxic conditions (0.5% O_2_) (Chamber Hypoxia Workstation CMVI300, IUL Instruments GmbH, Germany) (all growth factors: R&D Systems). Cell lysates were generated in RLT buffer (Qiagen, Hilden, Germany) and frozen at −80 °C until further analysis. Differentiated HBECs on 24-well trans-well inserts (Costar, Corning, NY) were stimulated basally with 5 ng/ml TGF-β1, control medium or kept under hypoxic conditions (0.5% O_2_). Cells were lysed in RLT buffer and lysates were frozen at −80 °C until further analysis.

### Animal experiments

Bleomycin-induced lung fibrosis models were performed using male C57Bl/6 J mice (9–12 weeks of age, Charles River Laboratories). For the initial Bleomycin challenge, animals were short term anesthetized (3–4% isoflurane, Abbott) and subsequently administered intratracheally with Bleomycin (1.0 mg/kg, Calbiochem, Billerica, MA, USA) in 0.9% NaCl aqueous solution (application volume 2 ml/kg body weight) or with a respective volume of 0.9% NaCl as vehicle control. For the intratracheal application, a 24G cannula connected to a 1 mL syringe was used. 14 days after the initial Bleomycin challenge, animals were killed by intraperitoneal injection of pentobarbital (400 mg/kg, Narcoren®, Merial, Germany). Prepared lungs were formalin-fixed and paraffin-embedded for subsequent histological analysis. All experiments were conducted in accordance with the German law on animal welfare (TierSchG) and have been approved by the Regierungspräsidium Tübingen.

### Gene expression analysis

RNA was isolated using RNeasy Kits (Qiagen, Venlo, Netherlands), followed by reverse transcription using the cDNA high capacity system (Life technologies, Carlsbad, CA) according to the manufacturer’s instructions. Gene expression was measured using the Taqman gene expression system and purchased assays on demand for the respective genes of interest (see list in Supplementary Methods). Fold changes were calculated using the ΔΔCt method^39^ and expressed either as ΔCt values, fold changes or percentage.

### Immunohistochemistry (IHC)

Normal and fibrotic lung tissue samples from the BLM-induced mouse model were prepared and Masson trichrome stained as described previously^27^. Human IPF and healthy lung tissue was purchased at Folio bioscience (Columbus, Ohio, USA) following the guidelines of the ethics committee. For IHC, lung tissue was processed using standard procedures, embedded in paraffin, and mounted on SuperfrostUltra Plus slides. Antigen retrieval was performed in citrate based buffer, pH 6.0. Antibodies: LOX: ab174316 (Abcam, Cambridge, UK) (dilution 1:300); LOXL1: NBP1-82827, (Acris Antibodies GmbH, Germany) (dilution 1:100), LOXL2: GTX105085 (GeneTex Inc., Irvine, CA) (dilution 1:500), LOXL3: LS-C165846 (LSBio, Seattle, WA) (dilution 1:500), LOXL4: sc-48731 (SantaCruz, Inc., Dallas, TX). Antibody dilutions were carried out in primary antibody diluent (Leica, Germany).

### Immunoblotting

Cells were lysed in RIPA buffer (Sigma, St. Louis, MO) containing Protease and Phosphatase Inhibitor Cocktail (Thermo Scientific, Rockford, IL). After centrifugation, protein concentration of the samples was determined using a BCA Assay (Thermo Scientific, Rockford, IL) and 10  ${\mu }{\rm{g}}$ total protein per lane were run on 4–12% Bis-Tris gels (Life technologies, Carlsbad, CA), electro blotted onto a PVDF-membrane (Millipore, Bedford, MA) and blocked with 5% w/v non-fat milk prior to antibody incubation. Antibodies (see immunohistochemistry) were diluted 1:1000 in blocking buffer and incubated at 4 °C overnight followed by standard imaging with HRP conjugates. GAPDH antibody was purchased from cell signaling technologies (14C10; #2118; Danvers, MS).

### Collagen crosslinking assay

Black µclear plates (Greiner bio one, Austria) were coated with human lung collagen (EPC, Owensville, Missouri, USA) at 4 °C overnight. Plates were washed with PBS once followed by incubation with either 20 nM or 100 nM recombinant LOXL2 or LOXL3 (R&D Systems, Minneapolis, MN) for 2 hours. The collagen reaction was stopped using fixative and free aldehyde residues were fluorescently labeled using the Aldehyde Site Detection Kit (KA1295, Abnova, Germany). FITC fluorescence was measured using EnVision and the respective Software.

### Preparation of conditioned media

HEK-293 cells were transiently transfected with plasmids encoding LOXL2-WT, LOXL2-ED or an empty control vector. The cells were incubated with the calcium-phosphate transfection mix for five hours, after which the medium was replaced and serum was reduced to 0.1%. The cells were then incubated for additional 72 h, to allow for expression and secretion of LOXL2 into the cell supernatant. After this time period, the medium was collected, centrifuged for 5 min at 200 × g and stored at 4 °C until further usage.

### Image analysis

FMT assay images were acquired using the IN Cell Analyzer 2000 imager (GE Healthcare, Freiburg) and analyzed using IN Cell Developer Toolbox 1.9.1 with a custom made analysis protocol. Therefore, 3 different target sets were defined: 1) “Nuclei_Locate” for nucleus detection with Nuclear as segmentation type in the DAPI channel. 2) “Cells” for cell detection with Object as segmentation type in the Cy2 channel. 3) “Fibril” for detection of fibrillary structures with Object as segmentation type in the Cy2 channel. Each target set was further refined using various different post-processing steps. The “Cell” and “Fibril” targets and the “Nuclei_Locate” and “Cell” targets were linked by “one-to-many” (Cell_Fibrils) and “one-to-one” (Nuclei_Cells), respectively. Finally, a composed target (“Fibril_in_cells”) set was created by a “one-to-one” linking of “Cell_Fibrils” and “Nuclei_Cells”. The mean number of fibrillary structures was measured in the linked target “Fibril_in_cells”.

### Statistical analysis

Statistical analysis was performed using GraphPad Prism 6 (GraphPad, La Jolla, CA). Differences between test groups were assessed by t-test or one-way ANOVA and Dunnet’s posttest for multiple comparisons.

## Electronic supplementary material

Supplementary information
