# Supplementary material for: Comparative analysis of lysyl oxidase (like) family members in pulmonary fibrosis
Source: Sci Rep. 2017 Mar 10;7:149. doi: 10.1038/s41598-017-00270-0 (PMC5428068; doi:10.1038/s41598-017-00270-0)
Supplement: Supplementary file 1 — Supplementary information [file 41598_2017_270_MOESM1_ESM.pdf]

SUPPLEMENTARY INFORMATION

Comparative analysis of lysyl oxidase (like) family members in pulmonary fibrosis

Verena Aumiller[1#], Benjamin Strobel[2#], Merrit Romeike[1], Michael Schuler[2], Birgit E. Stierstorfer[2] and Sebastian Kreuz[1]*

[1] Immunology & Respiratory Diseases Research, Boehringer Ingelheim Pharma GmbH & Co. KG, Biberach an der Riss, Germany

[2] Target Discovery Research, Boehringer Ingelheim Pharma GmbH & Co. KG, Biberach an der Riss, Germany

[#] *these authors contributed equally to this work*

* *Corresponding author*

Supplementary Results

Figure S1

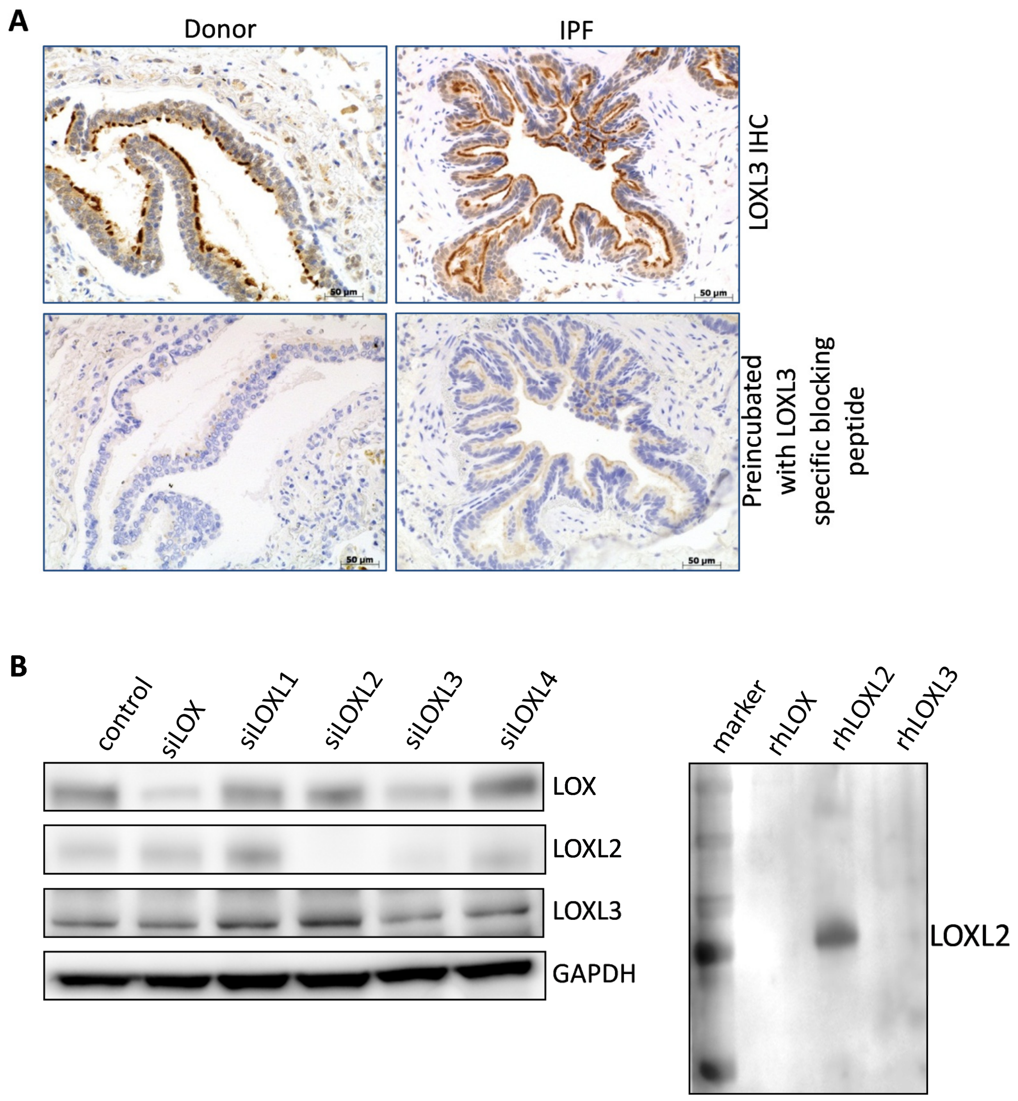

Figure S1: Antibody specificity. (A) Immunohistochemistry of LOXL3 in tissue specimen of transplant donors and patients (upper panel) with IPF after pre-incubation with LOXL3 specific blocking peptide (lower panel). (B) NHLF knockdown lysates (72 hours after siRNA transfection) were analyzed for LOX/L protein expression by Western Blot (left panel). LOXL2 antibody specificity was confirmed using recombinant human LOX, LOXL2 and LOXL3 protein (5 µg/lane) (right panel).

Figure S2

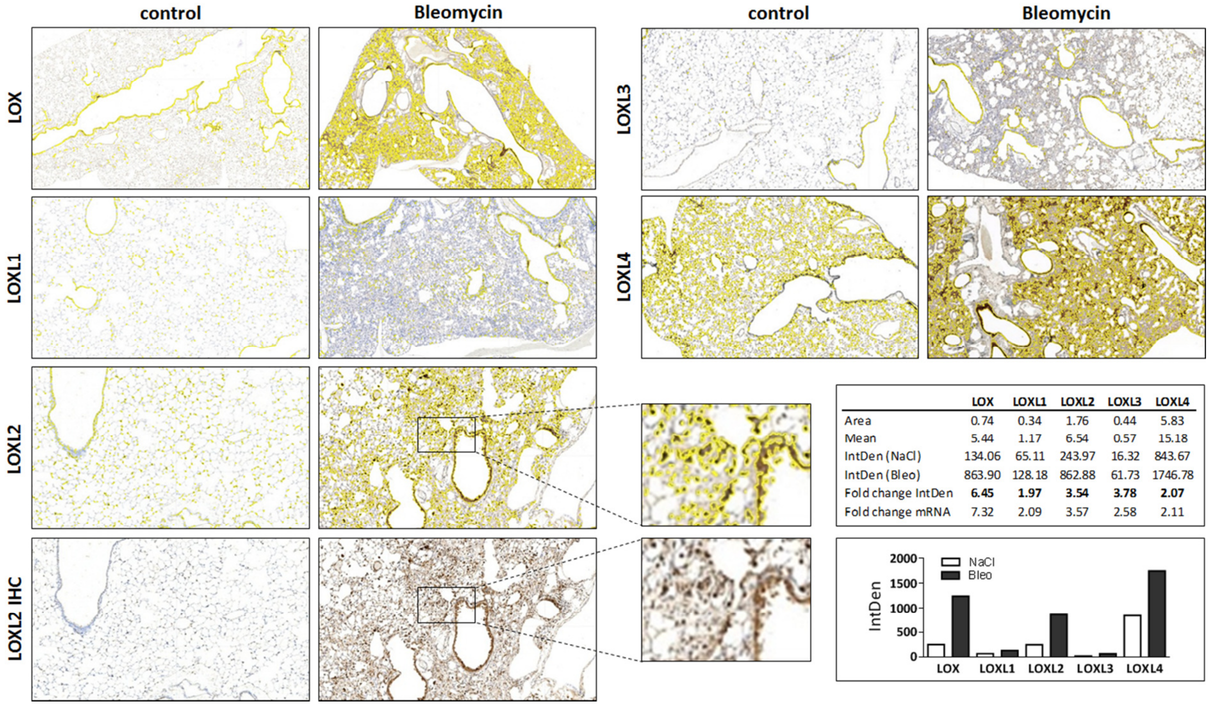

	LOX	LOXL1	LOXL2	LOXL3	LOXL4
Area	0.74	0.34	1.76	0.44	5.83
Mean	5.44	1.17	6.54	0.57	15.18
IntDen (NaCl)	134.06	65.11	243.97	16.32	843.67
IntDen (Bleo)	863.90	128.18	862.88	61.73	1746.78
Fold change IntDen	**6.45**	**1.97**	**3.54**	**3.78**	**2.07**
Fold change mRNA	7.32	2.09	3.57	2.58	2.11

Figure S2: IHC image-based analysis of LOX/L expression. Shown are sections of the IHC images presented in Figure 3. To assess LOX/L expression by computational image analysis, immunostained cells were selected using ImageJ software and the "Color threshold" function by defining thresholds for brightness and saturation (yellow selection in each image). Using the "measure" function, the integrated density (IntDen), i.e. the product of "Area" and "Mean Gray Value" in the selected area was determined. The IntDen values of the images from control (NaCl) and Bleomycin tissue sample images were used to calculate the "fold change protein" depicted in Figure 3d. Both, computationally analyzed and original IHC images as well as enlarged details are shown for LOXL2 to exemplarily illustrate the overlap between original staining and computationally selected areas. The table and graph in the lower right corner depict the "Area", "Mean" and "IntDen" measurement values used for calculation of the fold changes.

Figure S3

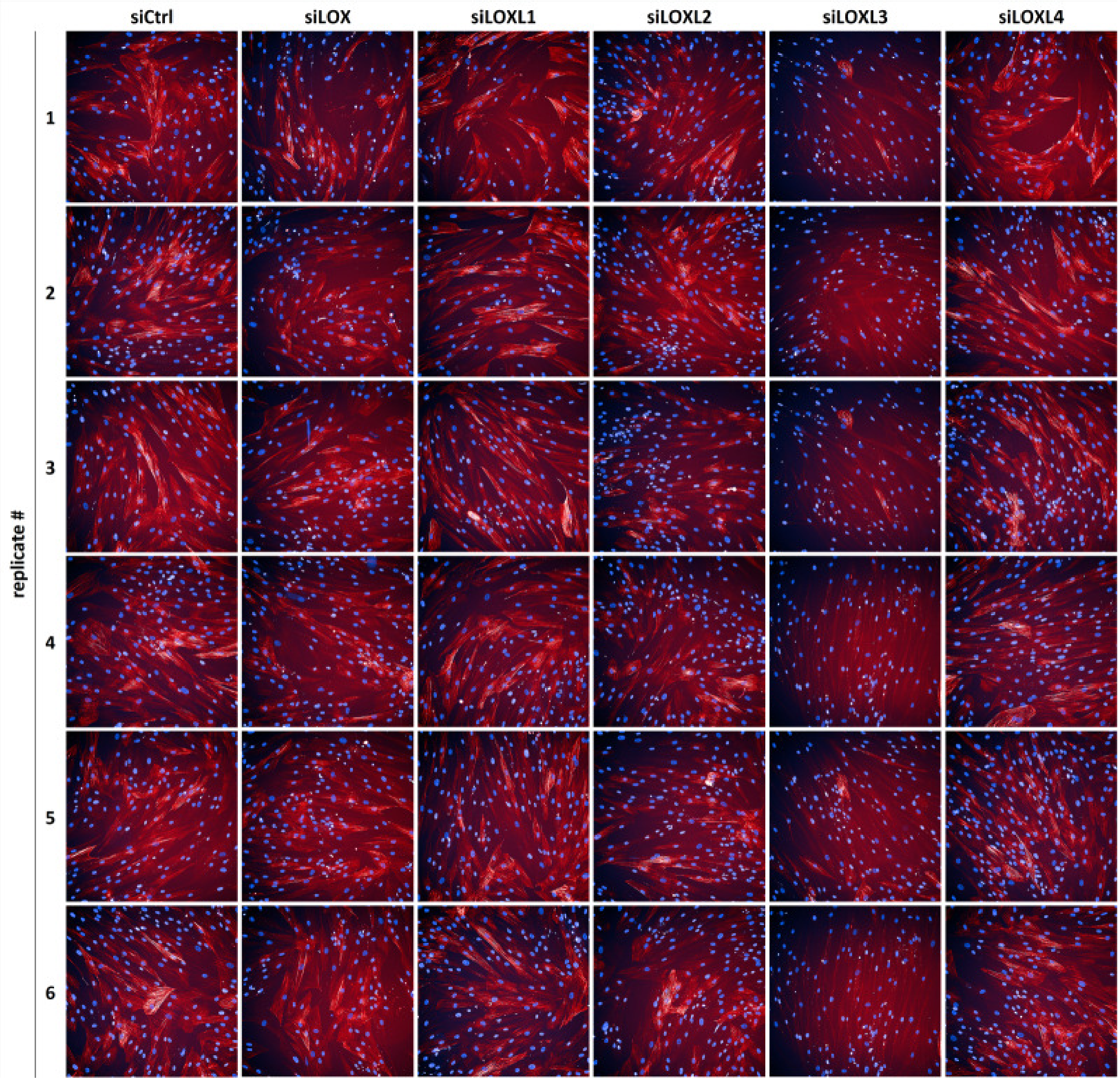

Figure S3: LOX/L knockdown leads to decreased smooth muscle actin production and stress fiber formation in NHLFs. Primary human lung fibroblasts (NHLFs) were transfected with 16.6 nM of control, LOX, LOXL1, LOXL2, LOXL3 or LOXL4 siRNA and stimulated with TGF-β1 for 72 hours. αSMA fibrils (red) per cell (DAPI, blue) were immunostained and imaged using high-content cellular imaging (see "Image analysis" in the methods section for details on αSMA fibril quantification). Six replicate images are shown per condition, representative of n= 3 independent experiments with 16 analyzed wells per condition each.

Figure S4

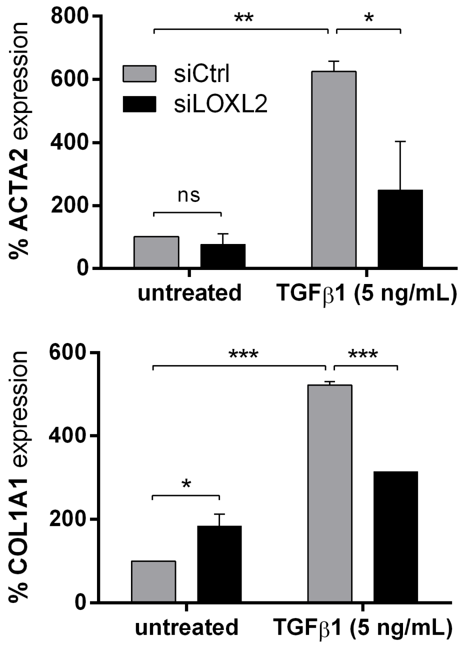

Figure S4: siRNA-mediated LOXL2 knockdown reduces ACTA2 and COL1A1 expression in TGFβ1-stimulated NHLFs. Primary human lung fibroblasts (NHLFs) were transfected with 16.6 nM of control or LOXL2 siRNA and stimulated with TGF-β1 for 48 hours. Gene expression of α-smooth muscle actin (ACTA2) and collagen 1α (COL1A1) were measured by qPCR. Mean ± SD from two biological replicates. *p<0.05, **p<0.01, ***p<0.001.

Figure S5

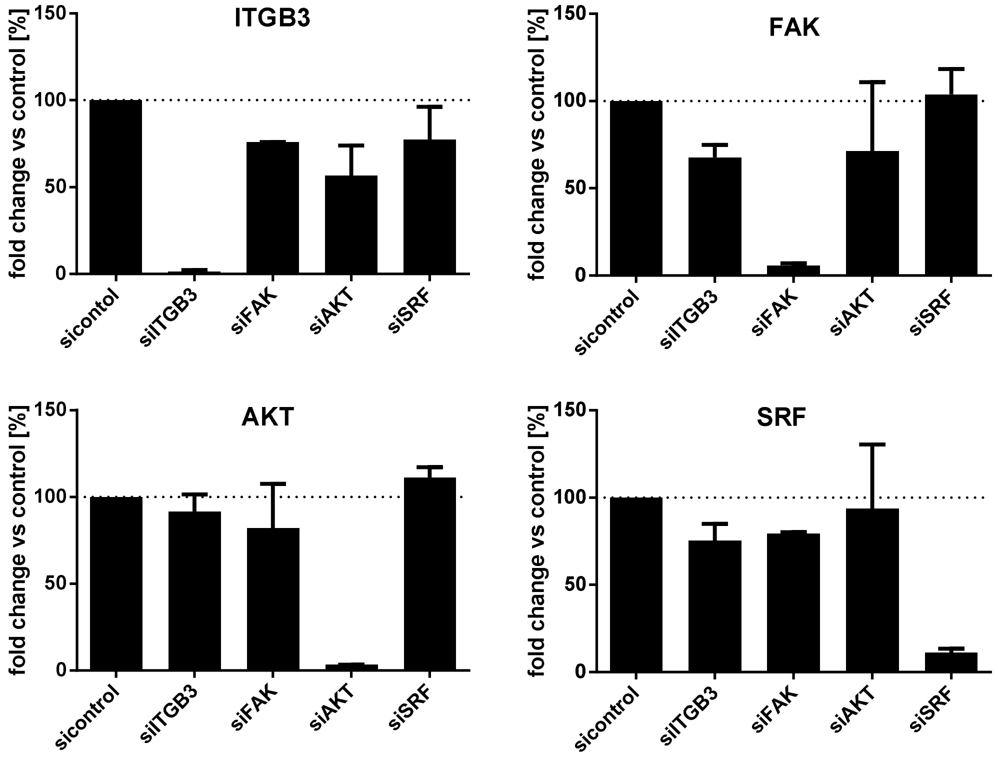

Figure S5: Knockdown specificity of siRNA pools. To exclude compensatory effects, gene expression of the FAK signaling cascade members ITGB3, FAK, AKT and SRF was analyzed following siRNA pool-mediated knockdown of each individual gene. 24 hours after transfection, mRNA levels for the individual FAK signaling cascade members were determined using Taqman gene expression assays. The data is depicted as % expression compared to control siRNA treatment and presented as mean ±SD of two to four independent experiments.

Figure S6

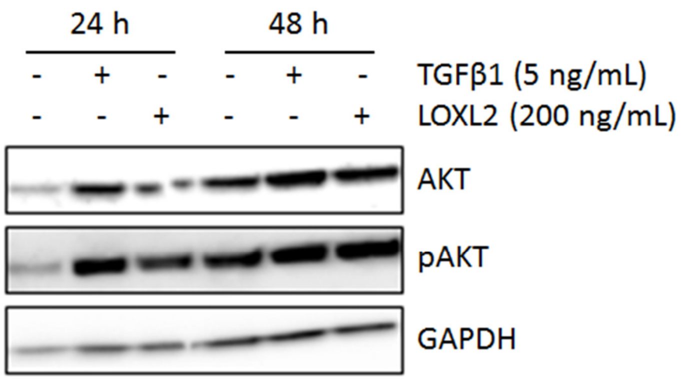

Figure S6: LOXL2 stimulation increases AKT phosphorylation. NHLFs were stimulated with either TGFβ1 or recombinant human LOXL2 (R&D systems) for 24 or 48 h, followed by cell lysis and Western blotting for total AKT, phospho-AKT (pAKT) and GAPDH, respectively.

Supplementary Methods

**List of siRNA pools used:**	GE Dharmacon catalog numbers**:**
siGLO RISC-Free® Control siRNA	D-001600-01-05
ON-TARGET plus Human LOX (4015) siRNA –SMARTpool	L-009810-00-005
ON-TARGET plus Human LOXL1 (4016) siRNA –SMARTpool	L-004645-01-0005
ON-TARGET plus Human LOXL2 (4017) siRNA –SMARTpool	L-008020-01-0005
ON-TARGET plus Human LOXL3 (84695) siRNA –SMARTpool	L-008021-00-0005
ON-TARGET plus Human LOXL4 (84171) siRNA –SMARTpool	L-008022-01-0005
ON-TARGET plus human PTK2 (5747) siRNA- SMARTpool	L-003164-00-0005
ON-TARGET plus human ITGB3 (3690) siRNA- SMARTpool	L-004124-00-0005
ON-TARGET plus human SRF (6722) siRNA- SMARTpool	L-009800-00-0005
ON-TARGET plus human AKT1 (207) siRNA- SMARTpool	L-003000-00-0005

List of Taqman gene expression assays used (Applied Biosystems catalog numbers)**:**

**LOX**	Hs00942480_m1
**LOXL1**	Hs00935937_m1
**LOXL2**	Hs00158757_m1
**LOXL3**	Hs01046945_m1
**LOXL4**	Hs00260059_m1
**pai1**	Hs01126606_m1
**ITGB3**	Hs01001469_m1
**SRF**	Hs00182371_m1
**FAK**	Hs01056457_m1
**AKT1**	Hs00178289_m1
**acta 2**	Hs00426835_g1
